# Effect of Multishell Diffusion MRI Acquisition Strategy and Parcellation Scale on Rich-Club Organization of Human Brain Structural Networks

**DOI:** 10.3390/diagnostics11060970

**Published:** 2021-05-27

**Authors:** Maedeh Khalilian, Kamran Kazemi, Mahshid Fouladivanda, Malek Makki, Mohammad Sadegh Helfroush, Ardalan Aarabi

**Affiliations:** 1 Department of Electrical and Electronics Engineering, Shiraz University of Technology, Shiraz 7155713876, Iran; maedeh.sadat.khalilian@etud.u-picardie.fr (M.K.); m.fouladivanda@sutech.ac.ir (M.F.); ms_helfroush@sutech.ac.ir (M.S.H.); 2 Laboratory of Functional Neuroscience and Pathologies (LNFP), University Research Center (CURS), University Hospital, 80054 Amiens, France; malek.makki@gmail.com; 3 Faculty of Medicine, University of Picardie Jules Verne, 80036 Amiens, France

**Keywords:** structural connectivity, rich-club organization, multiscale parcellation, multishell sampling, fiber tracking, DWI

## Abstract

The majority of network studies of human brain structural connectivity are based on single-shell diffusion-weighted imaging (DWI) data. Recent advances in imaging hardware and software capabilities have made it possible to acquire multishell (b-values) high-quality data required for better characterization of white-matter crossing-fiber microstructures. The purpose of this study was to investigate the extent to which brain structural organization and network topology are affected by the choice of diffusion magnetic resonance imaging (MRI) acquisition strategy and parcellation scale. We performed graph-theoretical network analysis using DWI data from 35 Human Connectome Project subjects. Our study compared four single-shell (b = 1000, 3000, 5000, 10,000 s/mm^2^) and multishell sampling schemes and six parcellation scales (68, 200, 400, 600, 800, 1000 nodes) using five graph metrics, including small-worldness, clustering coefficient, characteristic path length, modularity and global efficiency. Rich-club analysis was also performed to explore the rich-club organization of brain structural networks. Our results showed that the parcellation scale and imaging protocol have significant effects on the network attributes, with the parcellation scale having a substantially larger effect. Regardless of the parcellation scale, the brain structural networks exhibited a rich-club organization with similar cortical distributions across the parcellation scales involving at least 400 nodes. Compared to single b-value diffusion acquisitions, the deterministic tractography using multishell diffusion imaging data consisting of shells with b-values higher than 5000 s/mm^2^ resulted in significantly improved fiber-tracking results at the locations where fiber bundles cross each other. Brain structural networks constructed using the multishell acquisition scheme including high b-values also exhibited significantly shorter characteristic path lengths, higher global efficiency and lower modularity. Our results showed that both parcellation scale and sampling protocol can significantly impact the rich-club organization of brain structural networks. Therefore, caution should be taken concerning the reproducibility of connectivity results with regard to the parcellation scale and sampling scheme.

## 1. Introduction

The human brain connectome has greatly expanded our understanding of how cognitive processes emanate from a fundamental structural substrate [1]. In the past decade, structural connectivity analysis using graph metrics has been widely used to investigate the topological properties of brain structural networks derived from diffusion-weighted imaging (DWI) by modeling white-matter pathways connecting brain regions [2,3]. Many studies have focused on graph measures of network segregation (e.g., clustering coefficient and modularity) and measures of network integration (e.g., degree, characteristic path length and global efficiency) to investigate the small-worldness property of the human brain, exhibiting an optimal balance between the segregation and integration of information [2,4]. In recent years, there has been growing interest in the more complex topological properties of human brain networks. More specifically, the existence of a densely connected cortical “rich club” of hubs, playing a crucial role in global brain communication through short communication pathways, has been considered as the key characteristic of brain networks exhibiting a hierarchical structure [5]. It is suggested that any damage to cortical rich-club regions can cause large widespread disruption across large-scale brain networks with a significant impact on cognition [6,7,8,9].

To investigate the rich-club organization of large-scale brain structural networks, a brain graph is first constructed by gray-matter parcellation, in which parcels serve as nodes and links represent large-scale fiber tracts connecting nodes [10]. The brain networks can be represented at different spatial scales, from the microscopic (individual neurons) to macroscopic (brain regions) scales [11]. In general, brain connectivity analysis at multiple spatial scales can better capture the true hierarchical brain structure [11].

It has been shown that spatial scale and DWI acquisition protocols can significantly affect the behavior and topological properties of brain networks constructed using single-shell DWI data [2]. Advances in magnetic resonance imaging (MRI) have allowed white-matter fiber tracking at a high spatial resolution [12,13]. Recently, the NIH Blueprint for Neuroscience Research funded the MGH–USC consortium of the Human Connectome Project (HCP) to build the CONNECTOM scanner [14], which is capable of acquiring human diffusion MR data relying on contrasts from ultra-high b-values to resolve fine details of white-matter microstructure, a feature that does not exist in standard gradient MR systems, which are more sensitive to fast water diffusion and long-distance cortico-cortical connectivity at the macroscopic scale [15]. The broad range of b-values (1000, 3000, 5000 and 10,000 s/mm^2^) of the MGH–USC Adult Diffusion Dataset allows investigating patterns of structural connectivity by estimating slow water diffusion and shorter-range fibers, the main requirement to investigate the effect of diffusion parameters on brain structural networks and topology.

In this study, we performed graph-theoretical network analysis to quantify the extent to which the choice of parcellation scales and diffusion parameters (DWI sampling schemes with different b-values) could affect the organization of brain structural networks and topology. We further investigated the rich-club organization of brain structural networks at different nodal scales and DWI schemes by exploring whether the use of higher b-values could affect the estimation of short- to long-range white-matter streamlines.

## 2. Material and Methods

### 2.1. Processing Pipeline

**Figure 1:**
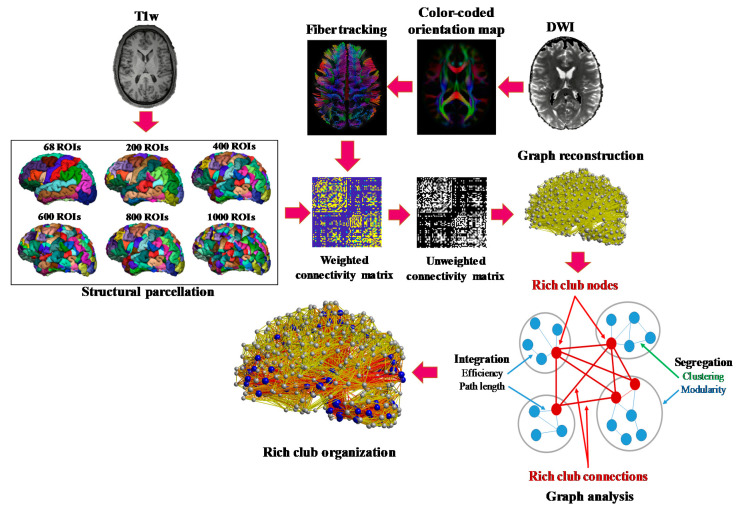
Processing pipeline for structural network analysis, including brain tissue segmentation, anatomical parcellation, single/multishell brain tractography and structural connectivity analysis using graph-theoretical metrics.

The processing pipeline includes brain tissue segmentation, anatomical parcellation, single/multishell brain tractography and structural connectivity analysis (Figure 1). The whole processing procedure is detailed in the following sections.

### 2.2. Imaging Data and Preprocessing

Thirty-five healthy adult subjects (19/16 males/females, 20–59 years old; mean age = 31.1 years old) were included in this study from the publicly available database of the Human Connectome Project (MGH–USC HCP database, https://ida.loni.usc.edu/login.jsp, accessed on 10 January 2020). Before data collection, informed written consent was obtained from all participants, and the experiments were approved by the institutional review board of Partners Healthcare [10].

All MR data, including anatomical (T1w and T2w) and diffusion-weighted (DW) images, were acquired on the 3T CONNECTOM MRI scanner. The T1w images were acquired with a 3D multi-echo magnetization-prepared rapid acquisition gradient echo (ME-MPRAGE) sequence and a 1 mm isotropic-voxel resolution. The T2w images were obtained with a T2-SPACE sequence at 0.7 mm isotropic resolution. The diffusion data were collected with a SE-EPI sequence with an isotropic resolution of 1.5 mm and parallel imaging. For data acquisition, four shells were selected at b = 1000, 3000, 5000 and 10,000 s/mm^2^ with 64, 64, 128 and 256 diffusion directions, respectively. One subject (MGH_1020) was excluded due to incomplete acquisition (482 volumes instead of 552 b = 10,000 s/mm^2^ volumes).

A non-DW image (b = 0) was acquired at the beginning of each run, as well as every 13 image volumes, yielding 552 volumes in total, including 512 DW and 40 non-DW volumes for each subject with a phase-encoding direction from anterior to posterior. The structural scans were corrected for geometric distortions. The concatenated diffusion data of all 4 b-values (552 image volumes) were also corrected for head motions and eddy current distortion [14,16,17]. In this procedure, the diffusion gradient table was adjusted for the rigid rotational components of the motion estimates [14]. The defacing and de-earing processes were finally carried out with FreeSurfer using face and ear delineation masks, respectively [14].

### 2.3. Structural Connectome Reconstruction

Deterministic fiber tracking was performed with DSI Studio (http://dsi-studio.labsolver.org, accessed on 30 February 2021) using the generalized q-sampling imaging (GQI) method [18]. To investigate the effect of sampling scheme on the organization of brain structural networks, four single-shell (with b-values of 1000, 3000, 5000 and 10,000 s/mm^2^) and multishell (with b-values of [1000, 3000] s/mm^2^, [1000, 3000, 5000] s/mm^2^, [3000, 5000, 10,000] s/mm^2^ and [1000, 3000, 5000, 10,000] s/mm^2^) diffusion schemes were used. The optimal value of the diffusion sampling length ratio was chosen for each scheme such that the fiber-tracking algorithm could resolve crossing fibers in crossing regions (e.g., in lateral corpus callosum) and also correctly estimate fiber directions in noncrossing regions (e.g., in the mid-corpus callosum).

Using each of the eight sampling schemes, a deterministic fiber-tracking algorithm was used to generate 1,000,000 streamlines for each subject by performing random seeding within the entire white-matter volume. Quantitative anisotropy (QA) was calculated for the orientation distribution function (ODF) peak in each voxel. QA enables direction-specific thresholding during tractography and is therefore less susceptible to partial volume effects and noise [19]. The angular threshold and step size were set to 45° and 0.75 mm, respectively [20]. The fiber trajectories were smoothed by averaging the propagation direction with a percentage of the previous directions, randomly selected from 0% to 95%. Tracks shorter than 30 mm or longer than 300 mm were discarded. The lower bound was set in DSI Studio to eliminate fiber fragments [21]. Finally, topology-informed pruning [22] was applied with two iterations to remove false connections.

For multiscale parcellation, the T1w anatomical data were first partitioned into 68 structurally defined regions using FreeSurfer (http://surfer.nmr.mgh.harvard.edu, accessed on 24 February 2021) according to the Desikan–Kiliany atlas [23]. Finer brain parcellations were then generated with approximately equal-sized regions at five parcellation (nodal) scales involving 200, 400, 600, 800 and 1000 parcels. We used FLIRT from FSL [24] to coregister the parcellations to the diffusion space. For each subject, this procedure was used to determine the optimal affine transformation between the T1w data (in which the parcels were based) and the b0 volume using the nearest neighbor cost function. We applied the resulting transformation to register the parcellations to the diffusion space.

#### 2.3.1. Network Construction

For each subject, forty-eight structural brain networks were constructed using the single- and multishell schemes at six nodal scales (i.e., 68, 200, 400, 600, 800, 1000) using DSI Studio (http://dsi-studio.labsolver.org, accessed on 30 February 2021). Each structural network W was represented by an N × N matrix, where N is the number of parcels (nodes), and each entry *w_ij_* represents the number of reconstructed streamlines connecting nodes *i* and *j*. For structural connectivity analysis, an adjacency (binary) matrix U was then constructed from each weighted network by a threshold defined as 0.1% (default threshold in DSI Studio) of the maximum number of track counts in the matrix. This threshold was used to discard spurious connections that were potentially influenced by noise. For the rich-club analysis, a backbone structural connectivity matrix (U_B_) was computed for each sampling scheme and nodal scale using the method described in [25]. In the backbone matrices, an entry of 1 between each pair of parcels indicated a structural connection between them in at least 75% of the subjects, and 0 otherwise.

#### 2.3.2. Topological Properties

To quantify the effect of the parcellation scale and DWI scheme on the topological properties of the unweighted structural networks, five graph metrics, including small-worldness, clustering coefficient, characteristic path length, modularity and global efficiency (see [10] for a brief description), were computed from each connectivity matrix for the different nodal scales and sampling schemes using the Brain Connectivity Toolbox implemented in Matlab (MathWorks, Inc., Natick, Massachusetts, United States) [26]. The small-worldness of each graph *G* was evaluated by computing the σ-ratio defined as:

(1) $$\sigma =\frac{\gamma }{\lambda }\;\gamma =\frac{C_{G}}{C_{R}}\;\lambda =\frac{I_{G}}{I_{R}}$$

where *C_G_* and *I_G_* denote the average clustering coefficient and the average path length of the graph, respectively, and *R* represents a “random” graph, which was constructed for the graph using the Erdős–Rényi (ER) model, in which both the graph and the random graph had the same average nodal degree [2]. For R, the values of *C_R_* (average clustering coefficient) and *I_R_* (average path length) were estimated as follows:

(2) $$C_{R}=\frac{d}{N}{\;I}_{R}=\frac{log\left(N\right)}{log\left(d\right)}$$

where *d* denotes the average nodal degree, and *N* is the number of nodes. Based on the σ-ratio, G has the small-world property of γ > 1 and λ ≈ 1. These two conditions were reduced to a single test σ > 1. We also used Newman’s method to estimate the modularity for each connectivity matrix by maximizing the number of within-group edges and minimizing the number of between-group edges in order to subdivide each network into nonoverlapping delineated groups of nodes [27].

#### 2.3.3. Rich-Club Coefficient for Unweighted Networks

To investigate the rich-club organization of the backbone networks (U_B_) constructed for each sampling scheme and nodal scale, for each degree *k* varying from 1 to the maximum degree in the network, a rich-club coefficient Φ(*k*) was computed as follows [1]:

(3) $$\Phi \left(k\right)=\frac{2.E_{>k}}{N_{>k}\left(N_{>k}-1\right)}$$

where, after removing all nodes N with a degree less than k, E _> k_ and N _≥ k_ represent the number of connections between the remaining nodes in *U_B_* and the total number of possible connections between the remaining nodes if they were fully connected, respectively. For each backbone network, a normalized rich-club coefficient Φ_norm_(*k*) was then computed with respect to Φ_random_(*k*), computed as the average rich-club coefficient over *m* (herein 1000) random networks of equal size with similar connectivity distribution, generated by randomizing the connections of the network while keeping the degree distribution of the matrix intact [25]. For each network, a normalized coefficient Φ_norm_ greater than 1 over a range of *k* reflects the existence of a rich-club organization for the network. A high rich-club coefficient indicates that the hubs are well connected. In general, the choice of the k level is arbitrary and study dependent [1,25]. For illustrative purposes, we selected the *k* level in a way to have 20% of each network’s nodes ranked as rich club to identify the rich-club spatial distribution for each parcellation scale and imaging scheme. Brain regions exhibiting the rich-club property were also identified in the backbone brain structural networks constructed for each sampling scheme and nodal scale.

In each network, nodes and connections were further classified into rich-club/non-rich-club nodes and rich-club/feeder/local connections, defined as connections linking members of the rich club, rich-club nodes to non-rich-club nodes, and non-rich-club nodes, respectively [1].

### 2.4. Statistical Test

To assess the statistical significance of rich-club organization, permutation testing was used [28,29]. The distribution of Φ_random_(*k*) computed over 1000 random topologies yielded a null distribution of rich-club coefficients. For the range of *k* within the rich-club zones, it was tested whether Φ significantly exceeded Φ_random_ (averaged over the examined range of *k*) by assigning a *p* value computed as the percentage of Φ_random_ that exceeded Φ. At each parcellation scale, the topological properties of the unweighted networks constructed for different sampling schemes at the subject level were compared using the nonparametric Friedman test to investigate the significance (with *p* < 0.05) of differences between the networks.

## 3. Results

The average streamline length for the single- and multishell DWI schemes ranged between 65.6 (single shell with b = 1000 s/mm^2^) and 70.1 mm (three shells with b = 3000, 5000, 10,000 s/mm^2^) (Table 1). As shown, the average streamline length increased with b-values higher than 3000 s/mm^2^. The normalized QA followed an inverse trend.

**Figure 2:**
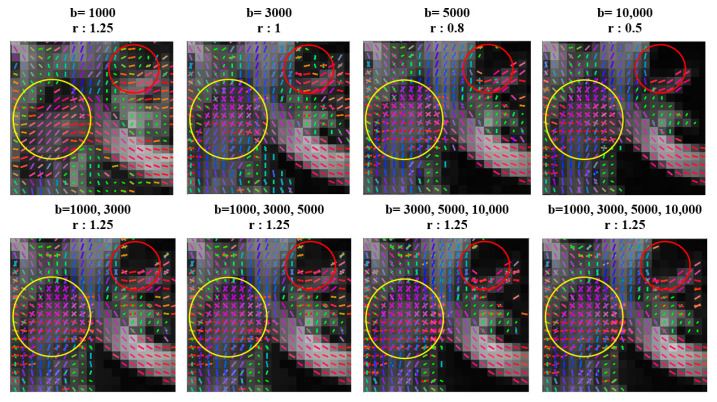
Effect of sampling schemes on fiber-tracking results at crossing regions (e.g., corpus callosum marked by yellow circles). Red circles show regions including false-positive fibers. The optimal values of the diffusion sampling length ratio (r) were determined for each DWI scheme using the optimization procedure specified in DSI Studio.

Figure 2 shows the effect of the sampling protocol on the tractography results of the corpus callosum (marked by yellow circles) containing crossing fibers. To obtain these results, the optimal value of the diffusion sampling length ratio (r) was determined for each sampling scheme using the optimization procedure specified in DSI Studio. As shown, the schemes with b-values higher than 5000 s/mm^2^ have better-resolved crossing configurations inside the voxels due to the higher angular resolution that can be achieved at higher b-values. In addition, visual inspection showed erroneous fiber tractography results (false fiber tracts marked by red circles) for the schemes with lower b-values (1000 and 3000 s/mm^2^). Overall, the inclusion of DW directions with higher b-values in the tractography procedure improved the tractography results at the locations where fiber bundles cross each other. Furthermore, our study shows that the three- and four-shell sampling schemes provided better tractography results for resolving crossing fibers with fewer false positives.

### 3.1. Graph Measures

**Figure 3:**
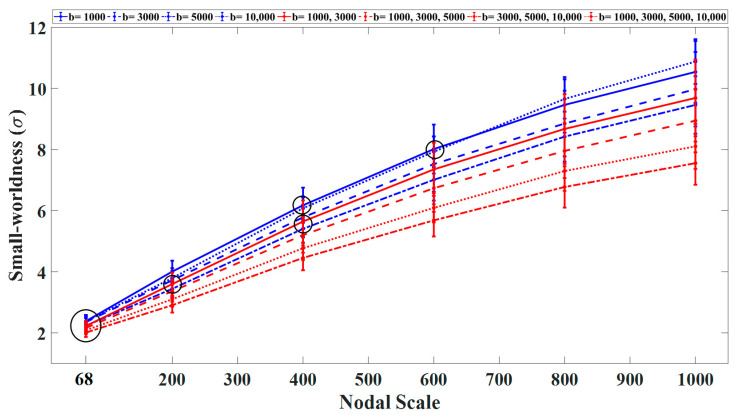
Average small-worldness curves (with S.D.) as a function of the nodal scale for single- and multishell sampling schemes. All statistical comparisons between the nodal scales and sampling schemes were significant (*p* < 0.05) with the exception of those marked by black circles.

Our results showed an increasing trend for the small-worldness curves (σ) with an increasing number of nodes for each DWI scheme (Figure 3). The networks constructed using the single-shell scheme with b = 1000 or 10,000 s/mm^2^ exhibited higher small-worldness in comparison with other b-values regardless of the nodal scale. In this category, the lowest small-worldness was obtained with b = 5000 s/mm^2^. Among the multishell imaging schemes, the networks constructed using the three-shell (b = 3000, 5000, 10,000 s/mm^2^) and two-shell (b = 1000, 3000 s/mm^2^) sampling schemes exhibited the highest and lowest small-worldness, respectively. Overall, the imaging schemes that included shells with b-values higher than 5000 s/mm^2^ exhibited significantly lower small-worldness. Moreover, the networks constructed at finer nodal scales displayed significantly (*p* < 0.05) higher small-worldness with larger confidence intervals across subjects in comparison with lower nodal scales.

**Figure 4:**
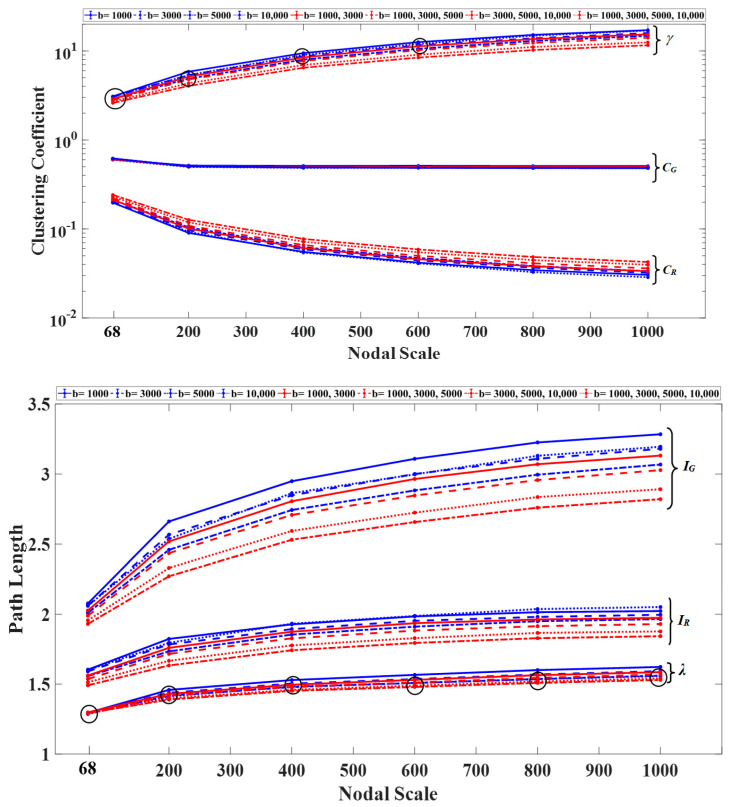
Average clustering coefficient (C_G_ and C_R_, shown on a logarithmic scale) and path length (I_G_ and I_R_) curves as a function of the nodal scale for the structural networks constructed for each DWI scheme and their equivalent random graphs R. The normalized clustering coefficient (γ) and path length (λ) curves are also shown for each nodal scale and sampling scheme. All statistical comparisons between the nodal scales and sampling schemes were significant (*p* < 0.05) with the exception of those marked by black circles.

To investigate whether the increase in small-worldness was due to an increase in clustering coefficient or a decrease in path length, we explored the trend of these metrics with an increasing number of nodes with respect to 1000 randomized networks generated for each structural network. As shown (Figure 4), both the normalized clustering coefficient and path length curves displayed upward trends. However, the normalized clustering coefficient showed a higher rate of increase with an increasing number of nodes, with lower values observed for the sampling schemes that included shells with b-values higher than 5000 s/mm^2^.

**Figure 5:**
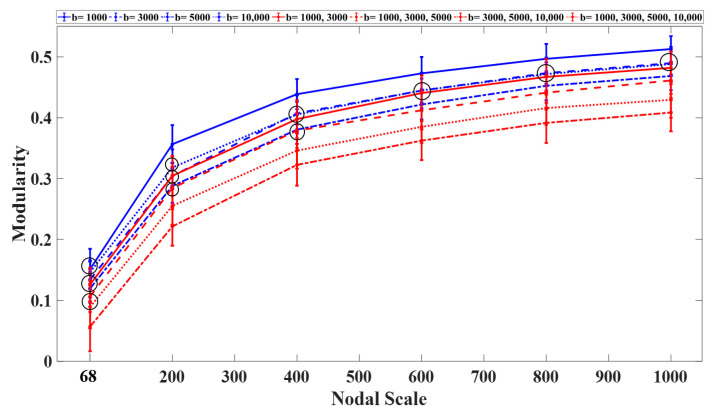
Average modularity curves (with S.D.) as a function of the nodal scale for sampling scheme. All statistical comparisons between the nodal scales and sampling schemes were significant (*p* < 0.05) All statistical comparisons between the nodal scales and sampling schemes were significant (*p* < 0.05) with the exception of those marked by black circles.

Modularity also showed an increasing trend with the nodal scale, demonstrating the tendency of the structural networks to form more communities at finer nodal scales, especially for the networks constructed using the single-shell schemes (Figure 5). Regardless of the nodal scale, the multishell sampling schemes that included shells with b-values higher than 5000 s/mm^2^ exhibited lower values of modularity in comparison with the single-shell or other multishell sampling schemes. Regardless of the sampling scheme, the statistical comparison showed significant differences (*p* < 0.05) between the modularity values computed for the networks at different nodal scales.

**Figure 6:**
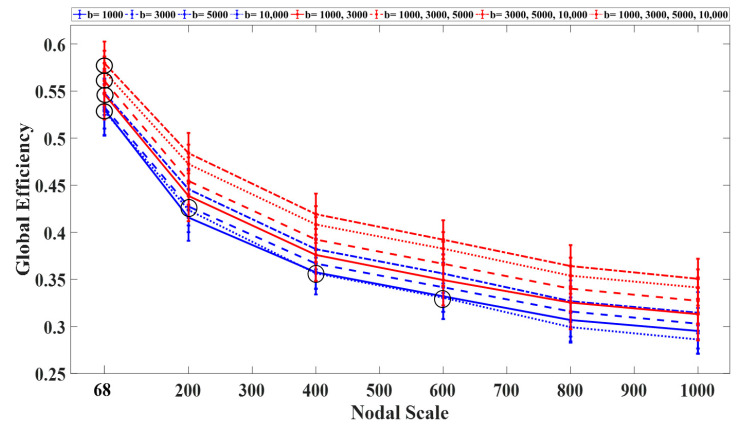
Average global efficiency (with S.D.) as a function of the nodal scale for each sampling scheme. All statistical comparisons between the nodal scales and sampling schemes were significant (*p* < 0.05) with the exception of those marked by black circles.

The global efficiency declined exponentially with the nodal scale (Figure 6). Among the single-shell schemes, the networks constructed using b-values of 3000 s/mm^2^ and 10,000 s/mm^2^ exhibited the highest and lowest global efficiency regardless of the nodal scale, respectively. Among the multishell sampling schemes, the networks constructed using two shells (with b = 1000, 3000 s/mm^2^) and three shells (with b = 3000, 5000, 10,000 s/mm^2^) exhibited the lowest and highest efficiency, respectively. Significant differences (*p* < 0.05) in global efficiency were observed between the single- and multishell schemes at different nodal scales. Overall, the inclusion of b-values higher than 5000 s/mm^2^ increased the global efficiency of the structural networks.

### 3.2. Rich-Club Organization of Structural Brain Networks

**Figure 7:**
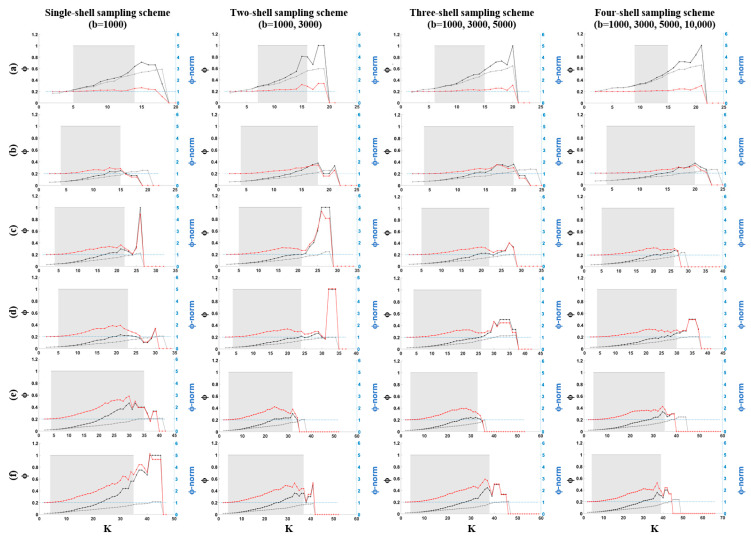
Rich-club curves (ϕ and ϕ_random_) as a function of degree (**K**) for each backbone structural network and its corresponding random networks for the single-shell (b = 1000 s/mm^2^), two-shell (b = 1000, 3000 s/mm^2^), three-shell (b = 1000, 3000, 5000 s/mm^2^) and four-shell (b = 1000, 3000, 5000, 10,000 s/mm^2^) sampling schemes at the nodal scales comprising (**a**) 68, (**b**) 200, (**c**) 400, (**d**) 600, (**e**) 800 and (**f**) 1000 nodes. The figures show the rich-club coefficient values for ϕ_norm_ (red), ϕ (black) and ϕ_random_ (light gray).

The rich-club coefficients ϕ(*k*) of the backbone structural brain networks (black lines in Figure 7) increased with the nodal scale for the single-shell and multishell sampling schemes. For simplicity, we only demonstrated the results for four sampling schemes. For the other schemes, similar trends of changes were observed. The normalized rich-club coefficient ϕ_norm_ (*k*) (shown in red) also displayed an upward trend. The intervals, in which the networks exhibited a rich-club organization, are shown in gray (*p* < 0.05, with permutation testing), with *k* levels within the range of 12 to 22 (Table 2). To compare the spatial distribution of rich-club nodes across different nodal scales and sampling schemes, we selected the *k* level in a way to have 20% of each network’s nodes ranked as rich club.

**Figure 8:**
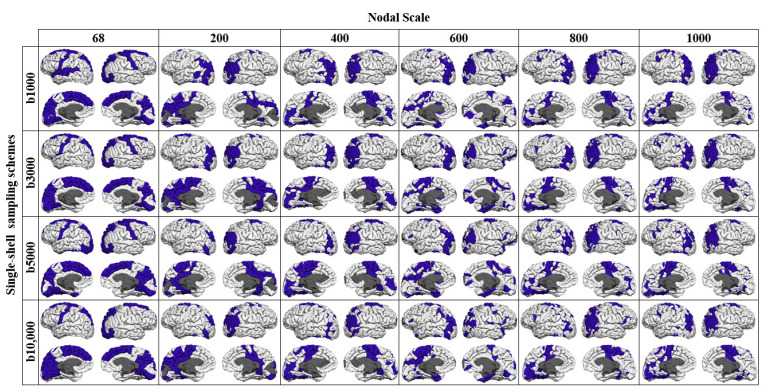
Spatial distribution of rich-club regions for the networks constructed using the single-shell sampling schemes at each nodal scale.

**Figure 9:**
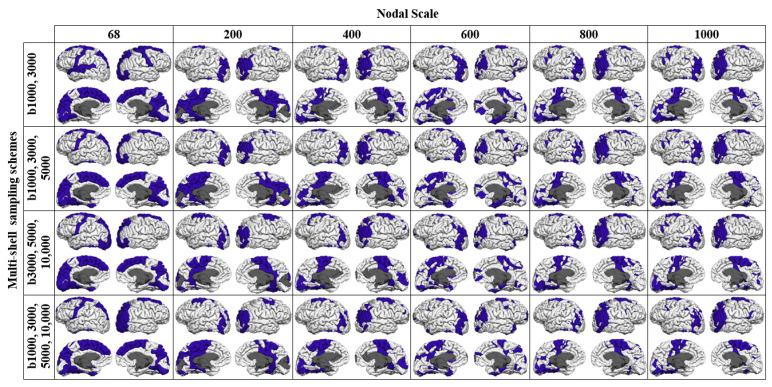
Spatial distribution of rich-club regions for the networks constructed using the multishell sampling schemes at each nodal scale.

As reported in Table 2, the *k* level increased at finer nodal scales. The inclusion of shells with higher b-values also slightly increased the *k* level. The proportion of rich-club connections increased with the nodal scale regardless of the sampling scheme used for connectivity analysis. An inverse trend was observed for feeder and local connections. The proportion of local connections decreased more slowly with k in comparison with that found for feeder connections across different sampling schemes. As shown (Figure 8 and Figure 9), rich-club nodes were mostly distributed in precuneus, superiorparietal, superiorfrontal, superiortemporal and precentral regions in both hemispheres of the brain. The spatial distributions of rich-club nodes were similar across different nodal scales that comprised at least 400 nodes especially for the multishell sampling schemes.

## 4. Discussion

In this study, we investigated the extent to which the brain structural organization was impacted by the parcellation scale (number of nodes) and the imaging protocols based on single-shell or multishell sampling schemes. For fiber tracking, we used GQI, shown to be an efficient method for solving complex crossing-fiber configurations. In this method, QA is calculated as an indicator of the population of spins in a particular direction. The QA-aided tractography has been shown to outperform the fractional anisotropy-aided tractography, especially in low SNR conditions [29]. At the group level, the topological properties and rich-club organization of the structural brain networks showed significant dependence on both the nodal scale and imaging scheme. The parcellation scale, however, affected more strongly the topology of the constructed brain structural networks. This finding is consistent with the results reported in [2,30].

With regard to the nodal scale, we calculated five graph metrics including small-worldness, characteristic path length, clustering coefficient, global efficiency and modularity, attributes widely used to characterize brain networks [2,25]. We found significant differences (with *p* < 0.05) between the values of the graph metrics derived from the single- and multi-shell-based brain structural networks across all parcellation scales. Increasing the number of nodes from 68 to 1000 resulted in significant increases (with *p* < 0.05) in small-worldness due to increases in the clustering coefficient. This trend of changes is probably due to the increase in the sparsity (ratio of the number of nodes-to-links) of brain structural networks [2]. We also found a significant increase in the sparsity of the networks with increasing the number of nodes from 68 to 1000. This seems to be the main reason for increasing the number of clusters of nodes located within the same neighborhood in large sparse networks [2]. To reduce the effect of the sparsity across different nodal scales, a proportional thresholding method, a strategy largely used in functional studies, can be used to adjust the sparsity rate across different networks according to the most sparse network (i.e., the network with the finest nodal scale). This approach is less employed in brain structural connectivity studies due to the fact that the brain structural networks are intrinsically sparse (as opposed to functional networks) [2]. To reduce the number of disconnected nodes in structural networks at finer scales, it has been suggested to increase the streamlined sampling rate and/or dilate gray matter [30]. This strategy might, however, increase the number of fibers falsely reconstructed due to the thicker cortex or higher streamline sampling rates, which might consequently change the networks’ topological properties. We further found that the networks’ modularity increased when the number of parcels increased. This might cause slower data transfer resulting in lower global efficiency, especially for larger brain networks. This is also reflected in the reduced efficiency observed in the brain structural networks at finer nodal scales.

With regard to the impact of the imaging protocol, the network metrics also showed dependence on the sampling scheme, although with a reduced effect compared to the nodal scale. Our results are in agreement with those reported by Zalesky et al. [2], who have shown differences between the topological attributes derived from the diffusion tensor imaging (DTI) and high angular resolution diffusion-weighted imaging (HARDI)-based brain structural networks in three healthy subjects. We further compared the topological properties of the single- and multi-shell-based networks. Overall, we found significant decreases (up to 17%) in small-worldness, modularity and clustering coefficient and a significant increase (by 11% on average) in global efficiency for the brain structural networks constructed using the multishell sampling schemes in comparison with the single-scheme imaging protocols, with larger differences observed at finer nodal scales. The connectivity measures obtained using the sampling schemes that included shells with b-values higher than 5000 s/mm^2^ were largely different from those obtained using the other single-shell or multishell imaging schemes, more likely due to longer white-matter streamlines generated with high b-values (Table 1). The presence of longer cortico-cortical connections in the brain network can significantly reduce the path length and therefore increase global efficiency. We further found lower modular values for the networks constructed by the single-shell and multishell sampling schemes including shells with high b-values (≥5000 s/mm^2^) across different nodal scales. In this case, the resulting networks exhibited higher efficiencies and tended to form fewer modules or communities connected through longer path lengths between high-degree nodes (hubs).

As also reported in [31], single-fiber situations can be reconstructed by all single-shell sampling schemes regardless of b-values. However, the inclusion of higher b-values (≥5000 s/mm^2^) can significantly improve the tractography results obtained using deterministic tractography at the locations where fiber bundles cross each other [20,32].

Finally, consistent with the findings of other studies [15,25,33], our results confirmed the rich-club organization for the brain structural networks, regardless of the imaging protocol and the parcellation scale used for network construction. The existence of a densely connected neural “rich club” of hubs has shown to play a crucial role in global brain communication through short communication pathways [1,15,25,33]. In networks exhibiting a rich-club organization, interactions between nodes are defined in a hierarchical manner from high-ranked rich-club connections to feeder or local connections. We, however, found that the spatial distributions of rich-club nodes were similar across the nodal scales that comprised at least 400 nodes. For the networks constructed using the multishell schemes, the rich-club cortical distribution showed patterns with significantly reduced spatial extent in comparison with the single-shell schemes. The proportion of rich-club connections also increased with increasing nodal scale regardless of the sampling scheme used for connectivity analysis (Table 2). An inverse trend was observed for feeder connections. The proportion of local connections showed a tendency to decrease with the nodal scale. In line with other findings [1], [15,25], we found that high-degree brain regions, including precuneus, superiorfrontal, superiorparietal and precentral regions, were identified as hubs in the structural networks across different parcellation scales and imaging protocols. It is suggested that any damage to cortical rich-club regions can cause disturbance across large-scale brain networks with a significant impact on cognition [8]. Due to their central role in information integration in large brain networks, rich club regions are also suggested to be generally vulnerable to neurological and psychiatric disorders [34].

Overall, our findings show that both the parcellation scale and sampling scheme can significantly affect the reproducibility of connectivity results. Therefore, comparison between different studies on structural brain network organization should be performed by taking into account the nodal scales and acquisition protocols used for connectivity analysis.

A limitation of our study is that we used deterministic fiber tracking that can generate many false-negative streamlines [35,36]. The deterministic fiber-tracking algorithms may also reconstruct physiologically unrealistic pathways [37]. This limitation can be overcome with probabilistic tracking, which incorporates anatomical information based on the distribution of possible directions into the tracking process. Another limitation concerns the dataset used in our study. The MGH–USC adult diffusion dataset was acquired using echo-planar pulse sequences, which are prone to image artifacts and distortions, which may affect structural connectivity results. Other pulse sequences such as field map correction, reversed gradient and PROPELLER DTI could be used to minimize the effect of susceptibility distortions on structural connectivity analysis [38,39]. These issues highlight an important focus for future investigations.

## 5. Conclusions

The main goal of our study was to investigate whether the topological properties of brain structural networks depend on the diffusion sampling scheme and the nodal scale. At the group level, the topological properties and rich-club organization of the structural brain networks showed significant dependence on both the nodal scale and imaging scheme. The parcellation scale, however, affected more strongly the topology of the constructed brain structural networks. Using the multiscale approach, we could evaluate the reproducibility of connectivity results across different parcellation scales, a critical step recommended in other studies [11]. Our results suggest that caution should be taken when comparing results from studies on structural network organization with regard to the parcellation scale and acquisition protocol. Further investigations should be performed to determine the optimal nodal scale and sampling scheme required to construct networks biologically closer to real brain structural networks [2].

## Figures and Tables

**Table 1:** Average streamline length (SL) and normalized quantitative anisotropy (nQA, mean ± S.D.) for the single- and multishell sampling schemes.

	Single-Shell Sampling Schemes				Multishell Sampling Schemes			
	b = 1000	b = 3000	b = 5000	b = 10,000	b = 1000, 3000	b = 1000, 3000, 5000	b = 3000, 5000, 10,000	b = 1000, 3000, 5000, 10,000
SL (mm) (mean ± S.D.)	65.6 ± 2.9	66.9 ± 2.6	69.2 ± 2.8	69.9 ± 1.9	66.9 ± 2.9	68.6 ± 2.7	70.1 ± 2.8	69.5 ± 2.6
nQA (a.u.) (mean ± S.D.)	0.142 ± 0.028	0.123 ± 0.017	0.112 ± 0.011	0.104 ± 0.022	0.125 ± 0.019	0.116 ± 0.013	0.106 ± 0.009	0.109 ± 0.009

**Table 2:** K level and proportion of rich club (RC), feeder (FC) and local (LC) connections linking rich-club members, rich-club nodes to non-rich-club nodes, and non-rich-club nodes, respectively, for the single-shell and multishell sampling schemes.

			Nodal Scale					
			68	200	400	600	800	1000
Single-shell sampling schemes	b = 1000	k level	12	12	14	14	15	15
		RC	15.2	17.2	25.6	29.9	35.9	40.4
		FC	48.1	39.2	33.6	29.2	26.6	22.8
		LC	36.7	43.6	40.8	40.9	37.4	36.8
	b = 3000	k level	13	14	15	16	16	17
		RC	15.9	20.2	25.2	28.2	34.3	36.8
		FC	45.3	33.6	32.2	31.9	27.1	26.5
		LC	38.9	46.2	42.5	39.8	38.6	36.6
	b = 5000	k level	15	15	16	16	16	17
		RC	13.3	19.6	24.5	26.7	31.4	35.1
		FC	50	33.9	32.1	33.2	30.8	26.9
		LC	36.6	46.5	43.4	40.1	37.8	37.9
	b = 10,000	k level	14	14	14	13	14	14
		RC	18.5	19.2	25.4	27.9	34.7	37.9
		FC	40.6	35.7	34.3	33.5	29.9	27.9
		LC	40.9	45.1	40.3	38.5	35.4	34.1
Multishell sampling schemes	b = 1000,	k level	14	14	15	16	17	17
	3000	RC	14.5	19.6	23.7	28	33.3	36.9
		FC	45.2	33.9	33	31.6	28.3	26.2
		LC	40.3	46.5	43.3	40.4	38.4	36.9
	b = 1000, 3000,	k level	15	16	17	17	18	19
	5000	RC	14.7	17.8	22.6	25.1	30.7	34.2
		FC	45.2	36.8	34.1	34.1	30	27.5
		LC	40.1	45.3	43.3	40.8	39.3	38.3
	b = 3000, 5000,	k level	15	18	18	19	21	22
	10,000	RC	13.3	16.8	21.4	25.4	30.5	32.8
		FC	45.8	38.3	35.1	32.6	28.9	28
		LC	40.9	44.9	43.5	42	40.6	39.2
	b = 1000, 3000,	k level	15	17	18	18	19	20
	5000, 10,000	RC	12.9	17.4	22.5	24.3	30.4	33.3
		FC	46.9	35.5	32.7	34.7	29.3	27.9
		LC	40.2	47.1	44.7	41.1	40.4	38.8

## Data Availability

The dataset analyzed in the current study is publicly available in the Human Connectome Project (HCP) repository (http://www.humanconnectomeproject.org/data, accessed on 1 January 2021).
